# Plating technique outcome evaluation in calcaneal fracture based on American orthopaedics foot and ankle score and Böhler-Gissane angle: A case series

**DOI:** 10.1016/j.ijscr.2022.107131

**Published:** 2022-04-29

**Authors:** Ananto Satya Pradana, Edi Mustamsir, Sulung Breilyan, Domy Pradana Putra, Krisna Yuarno Phatama, Mohamad Hidayat

**Affiliations:** Orthopaedics and Traumatology Department, Faculty of Medicine, Universitas Brawijaya-RSUD Dr. Saiful Anwar, Malang, East Java, Indonesia

**Keywords:** Calcaneal fracture, Plating technique, AOFAS score, Böhler angle, Gissane angle, Case series

## Abstract

**Introduction:**

Intraarticular Calcaneal fracture treatment nowadays is still up for debate. Surgical plating treatment is favorable because of the rapid healing process and better anatomical reduction despite the invasive intervention. Hence, clinical evaluation is needed to assess the quality-of-life index from foot and ankle by the American orthopaedics Foot and Ankle Society (AOFAS) score postoperatively. Then, the outcome evaluation of reduction in calcaneal plating of intraarticular calcaneal fracture with Böhler angle and Gissane angle to see if the calcaneal plating technique is a recommended treatment for the calcaneal fracture.

**Methods:**

We treated six patients from December 2020–July 2021 with a calcaneal fracture that underwent surgical plating, mainly by one surgeon. A calcaneal fracture is classified according to sanders classification. In this study, four patients are above the age of 40, and two are under 25. Pre-operative Böhler angle ranged from 8 to 65°, and Gissane angle ranged from 134 to 158°.

**Outcomes:**

Surgical plating was performed on all six patients. From clinical evaluation using the AOFAS score, we got satisfactory results on all patients who underwent calcaneal plating surgery. Three patients achieved excellent range outcomes with 95% and 99% of AOFAS Scores, and three patients reported AOFAS score good range outcomes with the lowest score of 88%. From the radiological outcome, most of the patient's Böhler and Gissane angles achieved normal value after surgical plating.

**Conclusion:**

The calcaneal plating technique gives better anatomical reduction depending on Bohler and Gissane angle. These results promise that anatomical reduction can improve clinical outcomes based on the AOFAS score. Thus, the plating method can be used effectively to treat an intraarticular calcaneal fracture.

## Highlights

• Limited studies reported the outcome method of “Plating technique” surgery for calcaneal fracture.
• Anatomical reduction achieved with Plating technique
• Satisfactory clinical outcome was concluded after Plating technique surgery for calcaneal fracture.

## 1 Introduction

Calcaneal fractures (CF) are the most common tarsal bone injury [1], [2]. CF occurs for approximately 2% of all fractures in the human body [3], [4]. The majority of calcaneal fractures are caused by high energy trauma, such as falls from a significant height [1], [5], [6], automobile accidents [4], and low energy trauma, e.g., sports injury [2]. Most calcaneal fractures occur in the young adult in their initial working years [4], [7]. This injury has a significant economic impact on both the patient and society due to the residual pain, time to mobilization, prevent 20% of patients from returning to work for up to three to five years following injury, prolonged hospital stay, and medical costs [7], [8].

The calcaneus is the biggest tarsal bone and is responsible for supporting the body [2]. As a result of the fractured deformity, there is frequently made abnormality of calcaneal height and varus and a wider heel [9]. A radiological examination is needed to evaluate the fracture line and compare it with the AO classification or sander's classification [3]. There are methods to measure abnormality of calcaneal height, such as The Bohler's and Gissane's angles [2], [9], [10].

The Bohler's and Gissane's angles of the calcaneum are important parameter characteristics in the diagnosis, treatment, and prognosis of calcaneal fractures [11]. Bohler angle is measured by the intersection angle between a line from the apex of posterior tuberosity to the apex of posterior facet and a line from apex of posterior facet to apex of anterior process on the lateral ankle X-ray [10]. Bohler's angle indicates fracture of the calcaneus with displacement changes. The angle of 20 degrees was strongly predictive of calcaneal fracture that normally ranges from 20° to 40° [10], [11]. However, Gissane's angles measurement made directly inferior to the lateral process of the talus, with the angle normal ranging between 120° to 140 [6]. Bohler and Gissene angle also can use as a significant marker of postoperative clinical outcome [9].

There is a discussion concerning how to treat calcaneal fractures associated with intraarticular displacement [1], [3], [7], [8]. Surgical management is an invasive intervention and could increase the infection risk [5] rather than conservative treatment [7]. However, surgical plating treatment is more favorable because of the rapid healing process, better anatomical reduction, and significantly shorter absence from work [3], [6], [12].

Nevertheless, there are limited studies on the relationship between calcaneal plating outcomes based on Bohler dan Gissane angle with AOFAS score. The AOFAS scores were divided as excellent (>90), good (80–90), fair (70–80), and poor (<70) [13]. Here we report an improved clinical outcome and radiological outcome after calcaneal plating surgery. This study aims to evaluate calcaneal plating outcomes for calcaneal fractures based on radiological outcomes and clinical outcomes. This work has been following PROCESS Guidelines [14].

## 2 Methods

This study was a prospective study. We underwent a plating technique on six patients with intraarticular calcaneal fracture. These patients underwent open reduction internal fixation with plating technique in Department of Orthopaedics and Traumatology, RSUD dr. Saiful Anwar, Malang between January 2020 and August 2021. We obtained medical data pre-and post-surgery from medical records and radiographic records. The mechanism of injury included falls from height, vehicle accidents, and other from injuries such as sports injuries. Gustilo and Anderson were used to classify soft tissue injury [15], and the sanders classification was used for calcaneal injury [16].

Inclusion criteria were: age over 17 years, open and/or close calcaneal fracture unilateral, displaced intraarticular calcaneal fracture from type IIA until IV by sanders classification, and soft tissue injury by Gustilo Anderson was included in these criteria. Exclusion criteria were: Age below 17 years old, bilateral calcaneal fracture, presented with any specific pathologic process such as a malignant or benign tumor, refused orthopedic management, suffered from major underlying conditions, including uncontrolled diabetes, kidney disease, immune system disease.

One experienced orthopedic surgeon performed calcaneal plating surgery. This study has obtained informed consent from all patients and was approved by the ethical committee of the Medical Research Faculty of Universitas Brawijaya.

### 2.1 Pre-operative management

**Fig. 1:**
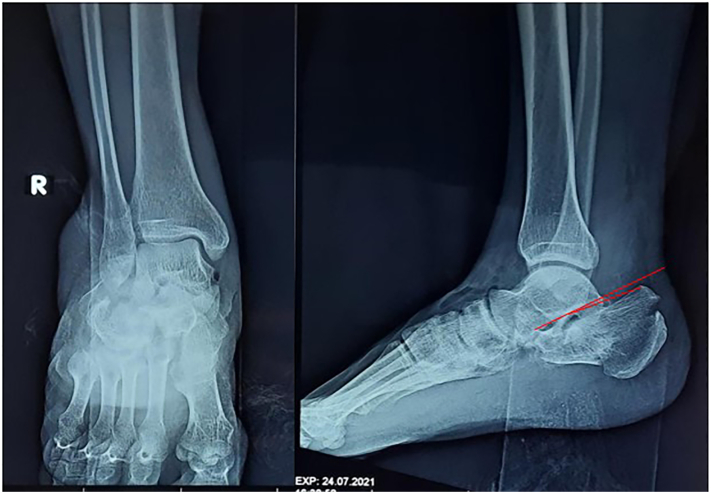
X-Ray pre-operative management with AP/Lateral view of left foot, with Gissane angle: 158° and Bohler angle: 12° (red line). (For interpretation of the references to colour in this figure legend, the reader is referred to the web version of this article.)

**Fig. 2:**
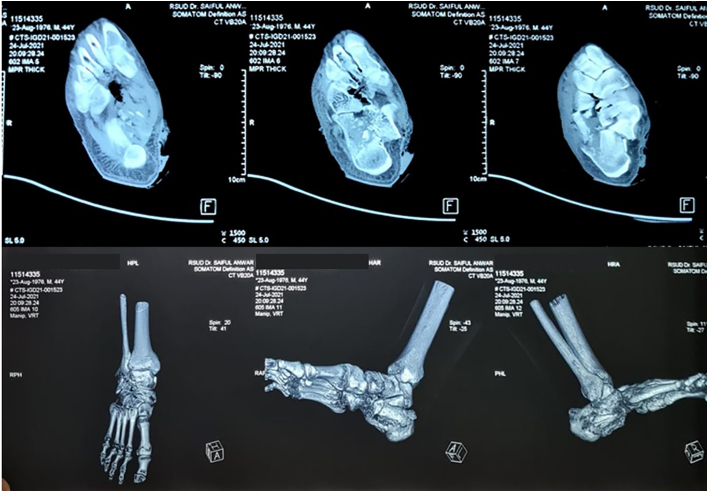
CT Scan of Calcaneal fracture pre-operative management.

Five patients came to ER after injury with a stable condition, while one patient needed improvement of condition before the surgery. We gave analgesics and antibiotics tailored to environmental exposure of the wound and standard wound treatment on the day of injury. These patients performed a radiographic evaluation with an anteroposterior (AP)/lateral ankle X-ray (Fig. 1), followed by a CT scan to ensure the calcaneal fracture configuration and determine the sanders classification of fracture (Fig. 2). The diagnosis of fracture calcaneus was based on the presenting imaging modality. Surgery was performed after any swelling was subsided.

### 2.2 Surgical technique

**Fig. 3:**
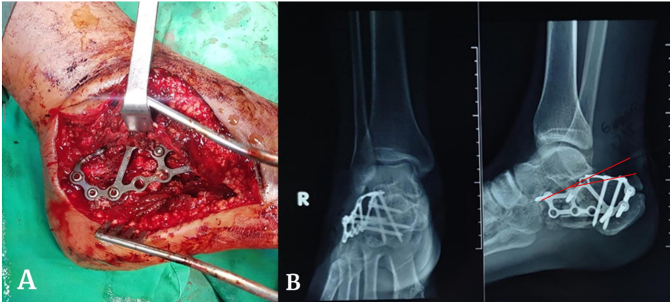
A. Calcaneal plating technique intraoperative on calcaneal fracture; 3B. X-Ray after operative management with AP/Lateral view of left foot, with Gissane angle: 135° and Bohler angle: 25° (red line). (For interpretation of the references to colour in this figure legend, the reader is referred to the web version of this article.)

The patient was placed in the lateral decubitus position on a radiolucent table with the fractured extremity facing upward. The lateral approach was performed with “L” incision used to reach the fracture in all patients. The subtalar joint was reduced under direct visualization after transecting the calcaneofibular ligament, then reduced the sustentaculum calcanei, anterior process, tuberosity, and then the posterior facet. Furthermore, Kirschner wires were also used to reduce and tentatively fix the calcaneal length and inclination. (Fig. 3A) Once the correction was achieved and confirmed with C-Arm, the internal fixation was performed using a plate and fixed using three or four leg screws. Furthermore, we performed backslab immobilization treatment for eight weeks.

### 2.3 Postoperative management

After surgery, all patients remained in the hospital for one week and were treated with an intravenous empirical antibiotic according to wound healing and pain reliever.

Rehabilitation started the two weeks postoperatively with active and passive range of motion exercises for the ankle and subtalar joint. The patient was restricted to be kept non-weigh bearing for 8–9 weeks depending on clinical and anatomical condition. All patients were routinely controlled, and in the 4th month after surgery, the patient stated that the pain in his foot decreased drastically, and he was gradually allowed to weight-bearing on his feet. After that, all the patients routinely control to our clinic's physical rehabilitation department for eight months.

### 2.4 Clinical and radiological assessments

Radiological evaluations were performed at four months and one year postoperatively. The anatomical reduction was evaluated with the Bohler and Gissane angle on the postoperative lateral ankle X-ray (Fig. 3B). Further, the clinical outcome was evaluated using American Orthopedic Foot & Ankle Society's (AOFAS) Score after one year of follow-up. A standardized scoring system consisted of nine questions covering three categories (Pain, alignment, and Function) with a maximum score of 100. The pain category was determined by asking about the patient's pain level, whereas the physician's examination decided the alignment category, and both the patient and the physician filled the function category.

### 2.5 Statistical analysis

Continuous data were expressed as means ± standard deviation. Paired *t*-test was used for comparison pre-and post-surgery and the relationship between variables. P < 0.05 were considered statistically significant. Statistical analysis was performed with Statistical Package for Social Science version 26.0 (SPSS 26.0).

## 3 Results

### 3.1 Characteristic patients

Our study consisted of five males and one female with a mean age of 38.3 ± 13.4 (Range 19–52 years old), and we followed up with all the patients until 12 months. The mechanism of injury in this study is caused by a fall from height in five patients and a motor vehicle accident (MVA) in one patient. In our study, three patients have an open fracture with Gustilo Anderson grade I, II, IIIA, respectively. And the other three patients have a closed fracture. Based on sanders classification, calcaneal plating surgery was recommended for calcaneal fracture classification sanders III or above. However, we performed calcaneal plating surgery on two patients with type II calcaneal fracture sanders because of displaced intraarticular configuration. (Table 1).

**Table 1:** Characteristic calcaneal fracture.

No	Gender, age (y)	Side and location wound	Open/close fracture	Gustilo Anderson	Sanders	MOI
1	M, 49 y	Left foot	Close	–	III	Fall
2	M, 52 y	Left foot	Open	I	IIIBC	Fall
3	M, 44 y	Right foot	Open	II	IIIAB	Fall
4	M, 19 y	Right foot	Close	–	IIA	MVA
5	F, 24 y	Left foot	Close	–	IIB	Fall
6	M, 44 y	Right foot	Open	III A	IIIAB	Fall

### 3.2 Clinical and radiological outcome

There was no surgical infection in all patients notated in 2 weeks during the initial follow-up. Then, radiology assessments were performed for four months and a one-year follow-up postoperatively. However, we only examined the outcome (clinically and radiologically) one year after surgery. X-Ray and CT scans were used to determine the height based on Bohler's and Gissane angles. In this study, it was found that there was a significant improvement in the AOFAS score (p < 0.000) between pre-and post-surgery after 1-year follow-up after calcaneal plating surgery. There were six patients with the poor clinical condition at the beginning who came with an AOFAS score range of 5–20, and after calcaneal plating surgery, there were three patients who reported clinical outcomes excellent scores (93,95, and 99), and three patients reported good clinical outcome (88,89 and 90) based on AOFAS score. Whereas, in terms of radiographic findings, Bohler's angle significantly reduced (p:0.0042) close to normal limits from the pre-operative range (8°–65°) to the postoperative range (14°–42°), and the Gissane angle also achieved significantly reduce close to normal limits (p:0.0025) postoperatively, with pre-operative range (130°–158°) to postoperative range (122°–142°). (Table 2).

**Table 2:** Clinical and radiographic pre and post calcaneal plating.

No	Gender, age (y)	AOFAS SCORE		BOHLER		GISSENE	
		Pre	Post	Pre	Post	Pre	Post
1	M, 47 y	5	90	52°	27°	144°	122°
2	M, 52 y	15	88	8°	14°	134°	136°
3	M, 44 y	10	89	65°	42°	155°	142°
4	M, 19 y	15	93	17°	34°	150°	140°
5	M, 44 y	15	95	12°	25°	158°	135°
6	F, 24 y	20	99	13°	27°	145°	135°
	Sig	0.000		0.042		0.025	

## 4 Discussion

Surgical management of calcaneal fracture along with intraarticular dislocation is still debatable [1], [7]. Consequently, in previous studies, variety management influences the outcome reported. A study conducted by Gusic, 2015 compared three surgical methods for calcaneal fractures. He stated that the appropriate method for treating intraarticular calcaneal fractures is a surgical treatment using a calcaneal plate [3].

We performed internal fixation surgery on all patients with open and closed calcaneus fractures. As expected, all of the patients obtained the outcome was satisfactory. All patients achieved good anatomical correction and were close to the normal range in evaluating radiological outcomes. While, in the evaluation of clinical outcomes, all of them achieved excellent outcomes depending on AOFAS Score. Some previous studies reported relevant to our finding that in displaced or comminuted fractures correction with calcaneal plating, a much better functional outcome was reported after Bohler's angle was restored [1], [17].

This study did not compare operative with non-operative outcomes regarding the calcaneal plating technique. However, several previous studies have stated that for intraarticular calcaneal fractures, anatomical correction is better by using operative management than non-operative management because of better functional outcome [5], shorter absence from work [7], and restored to a normal range motion [1], [18]. In contrast, studies reported that operative management increased infection risk5, and it can also be a non-union [19], even though it is a rare complication.

A study by Lee, 2019 stated that Bohler angle was not appropriate for sole references after operative reduction [9]. Therefore, in our study, we used two different types of measurement (Gissane and Bohler angle) to confirm the diagnosis and determine the plan of action and degree of reduction achieved before and after surgery. A statistical correlation test between the two parameters was also determined to ensure the result. Paired *t*-tests show that calcaneal plating can significantly correct the Bohler and Gissane angle close to the normal limit postoperatively on six patients. It is relevant to the study reported by Long, 2016. He mentioned that the calcaneal locking plate provides excellent stability for calcaneal fractures, reduces to normal anatomical angle, and allows weight-bearing at an early stage without interfering with stabilization [20].

In this study, no surgical site infection was reported in all patients during the follow-up period. In our opinion, the risk of infection was lowered because we chose the right antibiotic and used internal fixation methods. These results were reflected in a systematic review by Spierings, 2019 who also found that antibiotics prophylactic and internal fixation have a low complication rate, while the highest risk of infection occurred during the usage of antibiotics prophylactic in external fixation methods [21].

Early rehabilitation was also carried out to provide comprehensive management of patients after surgery and evaluate whether early rehabilitation could improve the functional outcome of the patients. Interestingly, the AOFAS score was significantly increased after surgery during the follow-up period. This result is relevant to a study by park, 2021, who reported that the outcome after earlier rehabilitation are excellent and give better results after calcaneal management [22]. In his study, the early rehabilitations performed were an early range of motion and early weight-bearing using heel weight-off walking after two weeks postoperatively. A small angle of the Bohler and Gissane angle that corrected the normal value postoperatively greatly impacts the patient's functional outcome. That proves it almost all patients have an increase in the AOFAS score after surgery.

The strengths of this study were that all of the patients underwent plating surgery with only one operator. Therefore, it can minimize the risk of bias, then the result of this study can be considered by a surgeon if they meet with similar cases because this technique gives satisfactory outcomes to most patients. However, this study also has several limitations, such as the small sample in our study and the short follow-up period. Moreover, the authors cannot intensively follow up on all patients in a pandemic era. The open and closed fracture categories in this study could also be one of the confounding factors for the outcome of this study. Therefore, a further study focusing on open or close fractures with significant sample size is suggested.

## 5 Conclusion

After surgery, the calcaneal plating technique gives better anatomical reduction based on Bohler and Gissane angles. Not only anatomical reduction but also this technique can improve clinical outcomes based on the AOFAS score. Therefore, the plating method can be used effectively in the treatment of an intraarticular calcaneal fracture.

## Source of funding

None.

## Data availability

The research article data used to support the findings of this study are available from the corresponding author upon request.

## Ethical approval

This study has been reviewed and approved by the authors' Institutional Review Board.

## Consent

Written informed consent was obtained from the patient for publication of this case series and accompanying images. A copy of the written consent is available for review by the Editor-in-Chief of this journal on request.

## Registration of research studies

This case series is not “First in Man” study.

## Guarantor

Ananto Satya Pradana, MD

## Provenance and peer review

Not commissioned, externally peer-reviewed.

## CRediT authorship contribution statement

Ananto Satya Pradana: data collecting, data interpretation, writing the paper and editing.

Edi Mustamsir: conceptualization, writing original draft preparation, supervision.

Sulung Berlyan: data collecting, data interpretation, writing the paper and editing.

Domy Pradana Putra: writing the paper and editing.

Krisna Yuarno Phatama: conceptualization, writing original draft preparation, supervision.

Mohamad Hidayat: conceptualization, supervision.

## Declaration of competing interest

None.
